# The Influence of Basicity/Acidity of Lanthanum Systems on the Activity and Selectivity of the Transesterification Process

**DOI:** 10.3390/molecules29122857

**Published:** 2024-06-16

**Authors:** Daria Dendek, Mateusz Zakrzewski, Radosław Ciesielski, Adam Kedziora, Waldemar Maniukiewicz, Małgorzata Szynkowska-Jóźwik, Tomasz Maniecki

**Affiliations:** Department of Chemistry, Institute of General and Ecological Chemistry, Lodz University of Technology, Zeromskiego 116, 90-924 Lodz, Poland; daria.dendek@dokt.p.lodz.pl (D.D.); radoslaw.ciesielski@p.lodz.pl (R.C.); adam.kedziora@p.lodz.pl (A.K.); waldemar.maniukiewicz@p.lodz.pl (W.M.); malgorzata.szynkowska@p.lodz.pl (M.S.-J.); tomasz.maniecki@p.lodz.pl (T.M.)

**Keywords:** biodiesel, heterogeneous catalysts, transesterification process

## Abstract

The impact of heterogeneous catalytic systems, which are based on rare earth metals, on the properties of biodiesel produced via the transesterification process in a stationary reactor (autoclave) was thoroughly investigated. The physicochemical attributes, including the specific surface area, were analyzed employing the Brunauer–Emmett–Teller (BET) method. The basicity and acidity levels of the catalytic systems were evaluated through temperature-programmed desorption of ammonia and carbon dioxide (TPD-NH_3_, TPD-CO_2_), respectively. Furthermore, High-Performance Liquid Chromatography (HPLC) analysis facilitated the assessment of triglyceride conversion and the determination of methyl ester (FAME) selectivity within these processes. Our findings indicate that catalytic systems augmented with lanthanum showcased superior performance. A significant correlation was discerned between the conversion and selectivity to methyl esters and both the specific surface area and the acidity and basicity properties of the catalytic systems under study. These results underscore the crucial role that the physicochemical characteristics of catalytic systems play in optimizing the transesterification process, thereby enhancing the quality of the produced biodiesel. This study contributes valuable insights into the development of more efficient and effective biodiesel production methodologies, highlighting the potential of rare earth metal-based catalysts in renewable energy technologies.

## 1. Introduction

Due to the ongoing depletion of fossil fuel reserves and the adverse environmental effects associated with traditional fuels, extensive research efforts are continually being directed toward the quest for optimal alternative fuels [1]. Nonetheless, alternative fuels must satisfy specific criteria, including minimal adverse environmental impact, readily available and renewable resources, and natural origins [1,2].

The initial references to the utilization of plant oils as alternative fuels emerged in the early 1980s. During this period, more than 350 oil-bearing plant species were subject to comprehensive examination. Plant oils predominantly consist of triglycerides, accounting for 90–98% of their composition, alongside minor proportions of monoglycerides, diglycerides, free fatty acids (FFA), phospholipids, phosphatides, carotenes, tocopherols, and trace amounts of sulfur compounds and water. Triglycerides are essentially esters of glycerol and three distinct fatty acids, distinguished by variances in carbon chain length and double-bond numbers [3].

Despite their significant resemblances to conventional fuels, particularly diesel, these alternative fuels exhibit certain drawbacks, including notable viscosity, a polyunsaturated character, and limited volatility [4,5]. These inherent properties may be effectively mitigated via specialized biodiesel production methods. Such methods encompass microemulsions, thermal cracking (pyrolysis), dilution, and transesterification.

Microemulsions formed by blending plant oils with short-chain alcohols, as well as dilution with solvents, result in products characterized by lowered viscosity. However, it should be noted that products derived from these techniques may be associated with engine performance issues, specifically the generation of carbonaceous deposits. In contrast, thermal cracking yields products falling within several distinct classes, encompassing alkanes, alkenes, aromatic compounds, and carboxylic acids. Unfortunately, these products are rendered unsuitable due to their pronounced ash content, carbonaceous residues, and elevated pour points. Notably, only transesterification represents the technique capable of yielding products that most closely emulate the properties of traditional diesel fuels. Consequently, it stands as the preferred method among those previously enumerated [6,7,8,9,10].

The transesterification process involves the chemical disintegration of raw oil molecules into methyl esters, facilitated through the use of alcohol and a catalyst. An associated byproduct of this reaction is glycerin. The equation governing the transesterification reaction is depicted in Equation (1).



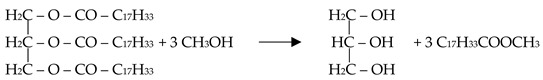

(1)


Biodiesel, derived from the transesterification reaction, possesses numerous merits, including biodegradability and non-toxicity, which have a minimal impact on atmospheric carbon dioxide levels. Biodiesel can be manufactured from biodegradable fractions or products, waste, or residues originating from biological sources in agriculture, encompassing both plant and animal materials, forestry, fisheries, and allied sectors, such as aquaculture. Moreover, it can be sourced from the biodegradable portion of industrial and municipal waste, spanning domains like waste management and wastewater treatment facilities.

The transesterification process is characterized by the use of a catalyst. There are three types of catalysts to consider: bases, acids, and enzymes. Each of these categories presents specific advantages and drawbacks, underscoring the pivotal role of catalyst selection. This choice not only influences the ultimate product but also exerts a significant impact on the reaction time and the overall cost of the transesterification process [11,12,13].

Enzymatic catalysts, distinguished by their high selectivity and the minimal or complete absence of byproducts, do, however, require substantially extended reaction durations and are notably more costly in comparison to alkaline or acidic catalysts. Consequently, from an economic perspective, enzymatic catalysts are the least frequently favored option for industrial biodiesel production [14].

Significantly, homogeneous catalysts are chosen more favorably for biodiesel production compared to those mentioned above. Both bases and acids have been categorized into homogeneous and heterogeneous catalysts. The alkaline catalysts most commonly employed in biodiesel production encompass potassium hydroxide (KOH), sodium hydroxide (NaOH), potassium methoxide (KOCH_3_), sodium methoxide (NaOCH_3_), and sodium ethoxide (NaOCH_2_CH_3_). These catalysts are prevalent choices in commercial biodiesel production due to the absence of water formation during the transesterification process. Furthermore, these catalysts exhibit significantly accelerated reaction rates (up to 4000 times faster) in contrast to acidic catalysts and do not necessitate the operation of transesterification at elevated temperatures or pressures, thereby reducing energy production costs. The resulting product achieved using these catalysts yields higher conversion rates compared to the utilization of acidic catalysts.

One noteworthy advantage of acidic homogeneous catalysts over their alkaline counterparts lies in their ability to prevent the formation of soap, a phenomenon associated with the presence of free fatty acids (FFA) in waste oils. The undesired formation of soap when reacting with alkaline catalysts impedes the separation of esters, glycerol, and water. Prominent examples of acidic catalysts include hydrochloric acid, sulfuric acid, sulfonic acid, and iron sulfate. Despite the recyclability and renewability these catalysts offer, as well as their ease of separation from the product, they are not as commonly favored as alkaline catalysts due to their lower reaction rates, necessitating the operation of reactions under high-temperature conditions, resulting in increased costs and heightened energy demands [14,15].

The relentless advancement in biodiesel production technology continually necessitates the exploration of alternatives to conventional homogeneous catalysts, with a growing focus on heterogeneous catalysts. These catalysts offer a plethora of advantages, increasingly positioning them as a preferred option in biodiesel synthesis. Notable benefits include the ease of separation from the final product, enhanced corrosion resistance, and environmentally benign attributes [15]. Furthermore, heterogeneous catalysts present several economic benefits in the transesterification process. Their reusability, owing to recyclability, absence of requisite additional purification stages, and potential cost-effectiveness as a substrate in biodiesel production, are particularly advantageous. Their versatility is underscored by the ability to tailor catalyst properties through appropriate combinations, facilitating desired outcomes in the production process [16]. This adaptability extends to the circumvention of the pre-esterification stage in cases where raw materials exhibit high free-fatty-acid content [17].

The current trend in heterogeneous catalysts gravitates towards those based on rare earth elements, lanthanides, alkaline earth metal oxides, supported alkaline earth metal oxides, and combinations of alkali and alkaline earth metals, alongside zeolites and other novel materials [17]. Among alkaline earth metal oxides, calcium oxide (CaO) has emerged as a particularly promising candidate. It facilitates reactions under mild conditions, demonstrates extended operational longevity, and importantly, ensures heightened transesterification activity [18]. However, a critical observation as per the study [19] is that CaO may rapidly lose its catalytic efficacy due to susceptibility to atmospheric H_2_O and CO_2_. This susceptibility, along with its proneness to leaching, detracts from its industrial appeal due to the implications on reusability.

In light of these challenges, attention has shifted towards rare earth metal oxides such as La_2_O_3_, CeO_2_, and Nd_2_O_3_. These oxides enhance thermal stability and catalytic efficiency, with La_2_O_3_ being particularly noteworthy for its combined basic and acidic properties and high catalytic activity [19]. A strategic approach involves the amalgamation of CaO with oxides of varying basicity or acidity to optimize the catalyst for the transesterification process. This strategy not only enhances basicity but also ensures the presence of necessary acid–base active sites. The robust tolerance of heterogeneous metal oxide mixtures to free fatty acids and moisture in vegetable oils is a crucial attribute in biodiesel production. The integration of CaO with MgO or ZnO, as reported in [19], has been shown to improve both the basicity and stability of the catalysts. La_2_O_3_, in particular, exhibits properties conducive to this process. Ultimately, the synergistic effect of combining an alkaline metal oxide (for the appropriate transesterification of triglycerides) with a rare earth metal oxide (to impart the necessary basic properties for free fatty acid esterification) paves the way for an optimized system, ideal for the synthesis of high-quality biodiesel [19].

## 2. Materials and Method

### 2.1. Preparation

The initial phase of the research involved synthesizing catalytic systems. The versatile properties of aluminum oxide (Al_2_O_3_) and its widespread applications are well-known and established in the literature. Various methods for obtaining aluminum oxide exist, with one such method being obtaining aluminum nitrate from an aqueous solution by precipitation, which was used in this work [20,21].

Ammonia was used as the precipitating agent. The precipitation process was carried out meticulously at 80 °C, gradually adding ammonia until the solution reached an alkaline pH, specifically in the range of 9–10. The precipitate was then aged overnight to improve its properties. The aged precipitate was then filtered under pressure, rinsed thoroughly with water until a neutral pH was obtained, and then dried in an oven for 24 h. The final product was calcined at 500 °C for 4 h.

A similar methodology was adopted for the synthesis of calcium oxide, although with adapted calcination parameters, specifically a temperature of 650 °C for 4 h. Sodium hydroxide served as the precipitant, the amount of which was adjusted to obtain a molar ratio of 0.5:1.0 with calcium nitrate.

These synthesized oxides were then used as the main components to prepare the desired catalytic supports. This was achieved using the impregnation method. An aqueous solution of calcium nitrate was added to the prepared alumina in such proportions as to establish the CaO/Al_2_O_3_ mass ratios of 50:50 and 70:30. This mixture was stirred in a magnetic stirrer for 2 h and then left for 24 h for impregnation. After impregnation, the aqueous solution was evaporated in a vacuum evaporator. The resulting precipitate was then dried for 24 h in a dryer and then calcined at 800 °C for 4 h.

The La_2_O_3_/Al_2_O_3_ support system was synthesized using a similar approach using an aqueous solution of lanthanum nitrate. This particular catalytic system was prepared with La_2_O_3_/Al_2_O_3_ mass ratios of 50:50 and 70:30.

When preparing the La_2_O_3_/CaO catalytic system, the procedure mirrored the above methods. The individual components were combined in proportions, resulting in a final product with a mass ratio of La_2_O_3_ to CaO of 30:70.

### 2.2. Methods and Instruments

To assess the physicochemical properties of the obtained catalytic systems, we utilized the Brunauer–Emmett–Teller (BET) method along with temperature-programmed desorption of NH_3_ and CO_2_ (TPD-NH_3_ and TPD-CO_2_) techniques.

For the surface area analysis of the sample, the Micromeritics AutoChem II+ apparatus (Ottawa, ON, Canada) was utilized. The sample (0.2 g) was purged with a helium stream while being heated to 350 °C within the apparatus. Subsequently, the sample was cooled to room temperature. Surface area measurements were conducted first in a cryogenic bath and then in a water bath.

For the assessment of catalyst acidity and basicity, we utilized the Micromeritics AutoChem II+ apparatus (Ottawa, ON, Canada). The procedure involved purifying the sample through one-hour heating at 600 °C under a continuous flow of helium. Subsequently, after cooling the sample for thirty minutes at 50 °C, it was appropriately saturated for acidity assessment using NH_3_ or for basicity assessment using CO_2_. The samples were then flushed with a helium stream at 100 °C. Following this step, temperature-programmed desorption (TPD) measurements were conducted in the temperature range of 100–600 °C, with a ramp rate of 20 °C/min, under a helium flow. The evolved NH_3_ and CO_2_ were recorded using a thermal conductivity detector calibrated based on the peak areas of known NH_3_ or CO_2_ signals.

The transesterification process was conducted using an autoclave, employing a mixture of rapeseed oil and methanol in a molar ratio of 1:8. However, an equal quantity of prepared catalytic systems, specifically 0.125 g, was utilized across all catalytic tests. Transesterification was carried out at temperatures of 100 °C and 150 °C for 1 h each. The resulting reaction products underwent High-Performance Liquid Chromatography (HPLC) (Agilent 1100, Japan) analysis to determine the methyl ester content.

HPLC analysis was performed to assess triglyceride conversion and determine FAME selectivity. An Agilent instrument equipped with a C-18 column was utilized. The eluent consisted of a mixture of 2-propanol and hexane in a 4:5 ratio along with methanol. Reaction products were analyzed using a Diode Array Detector (DAD) set at a wavelength of λ = 205 nm. Conditions of HPLC analysis are shown in Table 1 and parameters of HPLC are shown in Table 2. 

The morphological characteristics of catalytic systems were analyzed using scanning electron microscopy (SEM) conducted on an S-4700 electron microscope (Hitachi, Chiyoda, Japan), which was equipped with an energy-dispersive X-ray spectrometer (EDS) (ThermoNoran, Middleton, WI, USA).

The phase composition of catalysts after the reaction was investigated by X-ray diffraction (XRD) analysis on a Pro MPD diffractometer (PANanaliticsl, Almelo, The Netherlands) using CuK_α_ radiation in a 2θ range from 5° to 90°.

## 3. Results

### 3.1. Results of Conversion, Selectivity, and Cetane Number

Transesterification was conducted in a stationary reactor (autoclave) utilizing prepared catalysts. The reaction was carried out for one hour at two distinct temperatures, 100 °C and 150 °C. The biodiesel produced from this process was subjected to further analytical studies.

The initial stage of analysis involved the use of Gas Chromatography coupled with Mass Spectrometry (GC-MS). This method was employed to perform a qualitative and quantitative identification of the methyl esters present in the biodiesel sample. The biodiesel utilized for testing was obtained through a standard transesterification process employing a solid KOH catalyst in the presence of methanol [22].

The data acquired from this analysis were crucial for the subsequent phases of the research. Research provided insights necessary for determining the selectivity and conversion rates, as well as for calculating the cetane numbers of the various biodiesel samples. These parameters are vital for assessing the fuel quality and performance characteristics of the produced biodiesel.

The samples underwent High-Performance Liquid Chromatography (HPLC) analysis. The analytical protocol entailed the centrifugation of samples to eliminate sediments and reaction-related contaminants. This was followed by a 100-fold dilution of the liquid products in n-hexane. The prepared sample was then transferred to an analytical vial and introduced into the HPLC system using an autosampler.

The results obtained from the HPLC analysis were subsequently compared and correlated with those acquired from Gas Chromatography coupled with Mass Spectrometry (GC-MS) analysis. This comparative approach facilitated the precise assignment of methyl ester compounds to the respective peaks observed in the HPLC chromatograms, based on the quantification of methyl ester content in the product.

In the samples under study, the presence of three specific methyl esters was confirmed: hexadecanoic acid, methyl ester; 9-octadecanoic acid, methyl ester; and 11-eicosenoic acid, methyl ester. The conversion of the analyzed solutions was calculated using a predefined formula, allowing for a comprehensive evaluation of the transesterification process:

$$Con\left[\%\right]=\frac{{TG}_{in}-{TG}_{out}}{{TG}_{in}}\cdot 100\%$$

In this context, *TG_in_* represents the total integrated area under the peaks attributed to rapeseed oil, whereas *TG_out_* denotes the cumulative area of all triglyceride (*TG)* peaks present in the final product. The selectivity of the product was quantitatively determined by computing the ratio of the peak area of an individual product component to the aggregate area encompassing all identified product peak areas.

Furthermore, the cetane number, a crucial parameter for evaluating the efficacy of the biodiesel produced, was calculated. This was accomplished using a formula specifically devised for this purpose, which involves the summation of the product of the percentage of each ester and its respective share factor. The formula is presented below:

$$\phi_{B}=\frac{\phi_{1}\cdot A_{1}+\phi_{2}{\cdot A}_{2}+\cdots }{100\%}$$

where

*ϕ_B_* is the cetane number of the synthesized biodiesel,
*ϕ* is the cetane number of the methyl ester FAME,
*A* represents the molecular weight of the methyl ester FAME.

The cetane number coefficients for the relevant esters were calculated using the equation provided by Luis Felipe Ramírez-Verduzco, Javier Esteban Rodríguez-Rodríguez, and Alicia del Rayo Jaramillo-Jacob [23]:

$$\phi_{i}=-7.8+0.302\cdot M_{i}-20\cdot N$$

where

*ϕ_i_* is the cetane number of the methyl ester *FAME,*
*M_i_* represents the molecular weight of the methyl ester *FAME,*
*N* is the number of double bonds in a given methyl ester *FAME*

Selectivity is defined as the amount of each *FAME* to the amount of all occurring methyl esters in the sample tested. That is:

$$selectivity=\frac{amount\;of\;FAME}{amount\;of\;methyl\;esters}\cdot 100\%$$

The results of the aforementioned analyses are presented in Table 3.

The catalysts were evaluated in three distinct catalytic combinations: CaO/Al_2_O_3_, La_2_O_3_/CaO, and La_2_O_3_/Al_2_O_3_. Each catalytic system was investigated under two varying molar ratios, and the reactions were conducted at two distinct temperatures. Initially, the influence of temperature variation on the conversion efficiency of each catalytic system was assessed. It was observed that an increase in reaction temperature from 100 °C to 150 °C invariably enhanced the conversion rate across all systems.

Amongst the evaluated catalytic systems, the La_2_O_3_/CaO configuration exhibited the most favorable conversion outcomes. Alterations in the mass ratio within this system did not significantly impact the conversion rates of the tested samples. Conversely, the CaO/Al_2_O_3_ system was identified as the least effective catalyst, with changes in its mass ratio also yielding negligible variations in conversion rates.

Subsequently, the focus shifted to the selectivity parameter, defined as the ratio of the areas of methyl ester peaks to the overall peak areas within the sample. Here again, the La_2_O_3_/CaO system demonstrated superior performance. Notably, the La_2_O_3_/Al_2_O_3_ 70:30 and CaO/Al_2_O_3_ 50:50 samples exhibited the least favorable outcomes, with an absence of detectable Fatty Acid Methyl Esters (FAMEs). The La_2_O_3_/Al_2_O_3_ 50:50 system, however, showed the highest selectivity, but this was exclusive to the sample processed at 150 °C; lower reaction temperatures did not yield any methyl esters, underscoring the critical role of temperature in this parameter.

Directly correlated with selectivity is the cetane number, a crucial indicator of combustion quality in biodiesel. This metric was observed to increase in tandem with selectivity. The La_2_O_3_/Al_2_O_3_ 50:50 sample registered the highest cetane number. However, this does not conclusively denote it as the optimal catalytic system. The La_2_O_3_/CaO system emerged as a more stable and versatile option, consistently yielding high results irrespective of the mass ratio or temperature variations. This robustness renders the La_2_O_3_/CaO system highly adaptable for industrial applications, allowing for optimal reaction parameter adjustments to minimize costs while maximizing efficiency and output.

### 3.2. Results of Specific Surface Area, Alkalinity, and Acidity

The results obtained from BET, TPD-NH_3_, and TPD-CO_2_ measurements have been systematically compiled in Table 4. The BET analysis revealed that the La_2_O_3_/Al_2_O_3_ catalytic system exhibits the highest specific surface area among the systems studied. Notably, a correlation is observed wherein the specific surface area increases concomitantly with enhancements in both the cetane number and selectivity of the catalysts.

Furthermore, the TPD-NH_3_ and TPD-CO_2_ profiling demonstrated that the basicity and acidity of all the catalytic systems under investigation manifest through three distinct desorption stages. The analysis of the TPD profiles indicates the presence of three types of centers—weak, medium, and strong—in each system, of both acidic and basic characteristics.

A significant observation is the increasing ratio of basicity-to-surface area relative to acidity-to-surface area. This trend is consistent across the parameters of increased selectivity, cetane number, and the specific surface area of the respective catalytic systems. This finding underscores a potential interplay between surface properties and catalytic performance.

### 3.3. Results of Scanning Electron Microscopy (SEM)

The application of SEM is of paramount importance in the realm of material science, offering the capability to scrutinize the surface structures of samples with microscopic precision. This technique facilitates the acquisition of high-resolution images, thereby enabling the detailed assessment of both the morphology and the elemental composition of the materials under investigation. Such insights are indispensable for the advancement of research, as they contribute to the ongoing refinement of materials employed in further transesterification studies, thereby enhancing the overall efficacy and efficiency of these processes.

**Figure 1:**
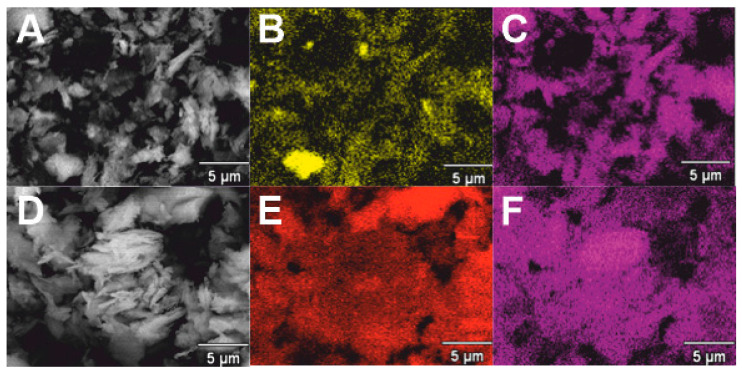
SEM images and maps of the elements’ distribution on the surface: (**A**) La_2_O_3_/Al_2_O_3_, (**B**) aluminum, (**C**) lanthanum, (**D**) La_2_O_3_/CaO, (**E**) calcium, and (**F**) lanthanum.

Figure 1A–D shows SEM photos and element distribution maps. SEM photos of both systems look very similar, i.e., it can be concluded that both catalytic systems have a similar structure. Analysis of the dispersion of elements in the La_2_O_3_/CaO and La_2_O_3_/Al_2_O_3_ catalytic systems showed their irregular and uneven distribution. It is worth noting that overlapping elements can be observed (Figure 1B,C,E,F), suggesting that aggregates may form in these areas.

### 3.4. Results of X-ray Diffraction

**Figure 2:**
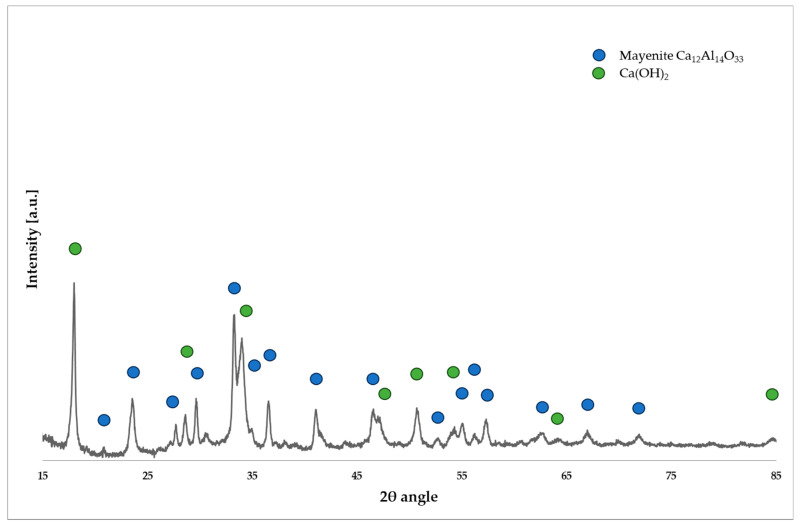
Diffractogram for the CaO/Al_2_O_3_ catalytic system.

Figure 2 illustrates a diffractogram for the CaO/Al_2_O_3_ catalytic system, displaying reflection angles from two phases: Ca(OH)_2_ and the mayenite structure. The findings indicate incomplete decomposition of the calcium catalytic precursor into calcium oxide, instead indicating the presence of its hydroxide. This deviation from the desired outcome may lead to moisture and carbonate absorption from the air, potentially diminishing the catalytic activity of the system. This is attributed to the relatively low calcination temperature of 800 °C, whereas the literature suggests a temperature of 850 °C or higher [23]. Additionally, the presence of the mayenite structure is deemed undesirable due to its potential reduction in the catalytically active surface area, consequently affecting the system’s activity adversely.

**Figure 3:**
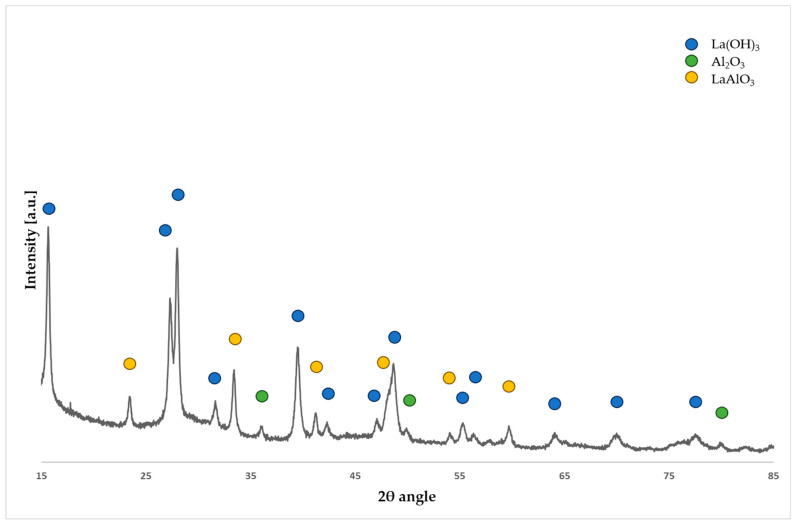
Diffractogram for the La_2_O_3_/Al_2_O_3_ catalytic system.

Figure 3 depicts the diffractogram of the La_2_O_3_/Al_2_O_3_ catalytic system, revealing the presence of structural phases such as Al_2_O_3_, La(OH)_3_, and LaAlO_3_. Analogous to the preceding system, the undesired formation of metal hydroxide, particularly La(OH)_3_, is evident. Furthermore, the occurrence of lanthanum aluminates exacerbates the decline in catalytic activity, as shown in the literature [23]. Despite the system’s potential for achieving high conversions and selectivity, mitigation strategies to prevent the formation of hydroxide and aluminate species are imperative for optimal performance.

**Figure 4:**
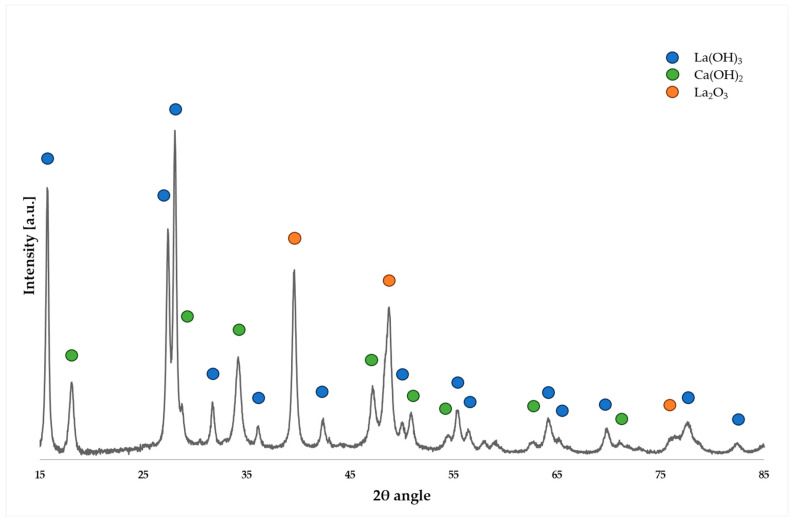
Diffractogram for the La_2_O_3_/CaO catalytic system.

The last diffractogram (Figure 4) illustrates the phase composition of the La_2_O_3_/CaO system. Notably, the system achieved a conversion rate of 91%, albeit with slightly lower selectivity to FAME compared to the La_2_O_3_/Al_2_O_3_ catalyst. This discrepancy may be due to the blocking of active sites by moisture and/or carbonates from atmospheric exposure, leading to the formation of hydroxide forms of calcium and lanthanum. These hydroxides were not completely converted into the corresponding oxides during the calcination process, likely due to insufficiently high calcination temperatures, which consequently adversely affects the catalytic activity [23]. The analysis of the diffractogram showed the presence of La(OH)_3_, Ca(OH)_2_, and La_2_O_3_ phases.

## 4. Discussion

Conversion and selectivity towards FAME are lower in the case of La_2_O_3_/Al_2_O_3_ catalytic systems compared to La_2_O_3_/CaO systems. This phenomenon can be attributed to the formation of aluminates on the surface of the catalyst supports in binary catalytic systems enriched with aluminum oxide. These structures exhibit reduced activity, consequently leading to inferior performance [24]. Furthermore, the disparity in results is influenced by the synergistic effect imparted by the addition of lanthanum oxide to CaO, as noted by Konokwan Ngaosuwan et al. [24]. This synergistic effect is contingent upon the presence of both Brønsted (-OH) and Lewis (-O) basic sites, thereby augmenting the rate of transesterification and the efficiency of FAME production [25]. It is noteworthy that La_2_O_3_ demonstrates both acidic and basic properties within a binary catalyst system. Observations reveal that in catalytic systems, oxygen sites correspond to Lewis basic sites responsible for facilitating the transesterification process, while metal ions are responsible for Lewis acidic sites, which promote the esterification process. According to research, interactions between La and Ca in such catalytic systems enhance the basicity and basic strength in La_2_O_3_/CaO systems compared to individual oxides of this catalyst, as corroborated in the literature [19]. Drawing from H.V. Lee’s findings, it is crucial to highlight that the increased basicity in mixed catalytic systems arises from the electron-donating behavior of La_2_O_3_, thereby reinforcing the interactions between reactant molecules and the catalyst surface [25]. The specific surface area increases with the rising content of lanthanum in catalytic systems, aligning with the existing literature [24]. The selection of an appropriate molar ratio of La_2_O_3_ significantly influences the reaction. The addition of a small quantity of lanthanum facilitates the dispersion of CaO on the catalyst surface and enhances the specific surface area [24]. The combination of La and Ca stabilizes CaO and imparts acidic–basic properties, which positively influence transesterification and esterification reactions, consistent with previous studies (Table 3) [25]. It is observed that by adjusting the proper ratio of La to Ca reagents, control over the basicity of the catalytic system is attainable, as the presence of these two oxides can generate robust basic centers, as documented in the literature [26,27]. Nonetheless, caution must be exercised to avoid the excessive addition of lanthanum oxide to the system, as an overabundance can lead to particle agglomeration on the surface of the investigated catalytic systems, evident in SEM (Figure 1B,C,E,F). Excessive La_2_O_3_ content can also impede selectivity to FAME, in line with findings from other researchers [19,24,25]. Nevertheless, the molar ratio of reagents is not the sole determinant of selectivity. The reaction temperature also plays a significant role, with the optimal selectivity to FAME achieved at 150 °C [25,27]. Selecting the appropriate temperature can enhance the reaction rate and improve oil–methanol miscibility, attributed to the higher temperature requirement for esterification catalyzed by La_2_O_3_-derived acidity in the binary La_2_O_3_/CaO catalytic system compared to base-catalyzed transesterification [25,28].

## 5. Conclusions

This research identified catalytic systems that show promising performance in the transesterification process carried out in a stationary reactor (autoclave) and deserve further research. The selection of appropriate reagents in these catalytic systems is a crucial factor because it allows the design of a catalyst that enables the production of biodiesel with optimal properties and, at the same time, is economically viable in industrial-scale applications. Physicochemical tests were employed to identify the catalytic systems that gave the most favorable results. In particular, it was noted that the introduction of lanthanum had a positive effect on the operation of the tested catalytic systems. Samples containing this element, such as La_2_O_3_/CaO 70:30, La_2_O_3_/CaO 50:50, and La_2_O_3_/Al_2_O_3_ 50:50, showed excellent conversion rates, selectivity, and cetane numbers. This enhancement is attributed to the stabilizing effect of lanthanum oxide on the catalytic system, leading to the desired production of methyl esters (FAME) and increased FAME content in the biodiesel samples. Moreover, studies have shown a relationship between the selectivity of catalysts and their specific surface area. We observed that an increase in surface area corresponds to a higher selectivity, indicating the importance of surface properties in the catalytic effect. This relationship is also consistent in tests of the basicity and acidity of catalytic systems. In conclusion, the research highlights the complex relationship between surface properties and catalytic performance in biodiesel production. These insights add to the knowledge of catalytic systems and their potential in industrial applications, highlighting the importance of a tailored catalytic system for optimizing biodiesel synthesis processes.

## Figures and Tables

**Table 1:** Conditions of HPLC analysis.

Optimized Gradient of Phase Used to HPLC Analysis	
Solvent A	methanol
Solvent B	2-propanol/hexane 4/5
Injection volume	5 μL
Column temperature	30 °C

**Table 2:** Parameters of HPLC analysis.

Mobile Phase Gradient			Flow Rate [mL/min]	Maximum Pressure [Ba]
Time [min]	Solvent A [%]	Solvent B [%]		
0.00	100.00	0.00	0.25	400.00
20.00	100.00	0.00	0.25	400.00
45.00	50.00	50.00	1.00	400.00
46.00	50.00	50.00	1.00	400.00
57.00	50.00	50.00	1.00	400.00
70.00	0.00	100.00	1.00	400.00
71.00	100.00	0.00	1.00	400.00

**Table 3:** Conversion and selectivity results for the obtained catalytic systems and cetane number of transesterification process products.

Sample	Temperature [°C]	Time of Reaction [h]	Conversion [%]	Cetane Number	Selectivity [%]	
CaO/Al_2_O_3_ 70:30	100	1	33	LACK	LACK OF METHYLESTERS	
	150		49	4	Hexadecanoic acid, methyl ester	2.12
					9-Octadecenoic acid, methyl ester	2.67
					11-Eicosenoic acid, methyl ester	0.47
CaO/Al_2_O_3_ 50:50	100	1	43	LACK	LACK OF METHYLESTERS	
	150		62	LACK	LACK OF METHYLESTERS	
La_2_O_3_/CaO 30:70	100	1	67	33	Hexadecanoic acid, methyl ester	12.84
					9-Octadecenoic acid, methyl ester	21.45
					11-Eicosenoic acid, methyl ester	13.48
	150		99	32	Hexadecanoic acid, methyl ester	19.41
					9-Octadecenoic acid, methyl ester	18.70
					11-Eicosenoic acid, methyl ester	9.67
La_2_O_3_/CaO 50:50	100	1	66	13	Hexadecanoic acid, methyl ester	4.87
					9-Octadecenoic acid, methyl ester	10.07
					11-Eicosenoic acid, methyl ester	5.06
	150		91	42	Hexadecanoic acid, methyl ester	17.78
					9-Octadecenoic acid, methyl ester	28.19
					11-Eicosenoic acid, methyl ester	15.82
La_2_O_3_/Al_2_O_3_ 70:30	100	1	68	LACK	LACK OF METHYLESTERS	
	150		74	LACK	LACK OF METHYLESTERS	
La_2_O_3_/Al_2_O_3_ 50:50	100	1	61	LACK	LACK OF METHYLESTERS	
	150		70	51	Hexadecanoic acid, methyl ester	22.19
					9-Octadecenoic acid, methyl ester	34.21
					11-Eicosenoic acid, methyl ester	18.45

**Table 4:** Test results for acidity, alkalinity, and specific surface area of the catalytic systems.

Sample	Temperature [°C]	Time of Reaction [h]	Surface Area [m^2^/g]	Basicity/Surface Area [µmol/m^2^]	Acidity/Surface Area [µmol/m^2^]
CaO/Al_2_O_3_ 70:30	100	1	7	1716	137
	150				
CaO/Al_2_O_3_ 50:50	100	1	9	962	99
	150				
La_2_O_3_/CaO 30:70	100	1	7	1594	232
	150				
La_2_O_3_/CaO 50:50	100	1	14	463	59
	150				
La_2_O_3_/Al_2_O_3_ 70:30	100	1	22	375	22
	150				
La_2_O_3_/Al_2_O_3_ 50:50	100	1	44	238	13
	150				

## Data Availability

The data presented in this study are available upon request from the corresponding author.
